# Correspondence

**Published:** 1908-08

**Authors:** Cora Frances Stoddard

**Affiliations:** Corresponding Secretary; Boston, Mass.


### Correspondence.

### The Scientific Temperance Federation.

Boston, Mass., July 9, 1908.

*Dr. F. E. Daniel, Editor Texas Medical Journal, Austin, Texas.*

Dear Sir: Yesterday on looking through the June number of the Texas Medical Journal, which came during my absence from home, I discovered the editorial quoted from *American Medicine* on the subject of tobacco and the Meylan investigation at Columbia. It would appear that *American Medicine* has done a triple injustice in its hasty conclusions from these experiments: First of all, to itself, as, so far as the writer has been able to learn, no authentic report of this work has yet been issued, and hence *American Medicine* has apparently placed itself in the position of drawing hasty and insufficiently founded conclusions in the very article in which it condemns such proceedings. Secondly, to Professor Meylan, possibly, judging from what he himself says in the letter quoted in my enclosed communication. And, thirdly, more serious than either of the foregoing, to the general public, which will easily be misled by the report to which *American Medicine* has thus given the stamp of its approval.

I am sure, therefore, that you will wish to give your reader the facts which I have stated in the enclosed letter to the editor of the Texas Medical Journal. Yours very truly,

Cora Frances Stoddard,
Corresponding Secretary.

Here follows the article:

### TOBACCO: A FEW IMPORTANT FACTS.

*Editor of Texas Medical Journal, Austin, Texas:*

The Texas Medical Journal for June quotes from a March number of *American Medicine* an article which it describes as "an attack upon what is termed 'popular prejudice' against the cigarette, and an attempt to discourage anti-tobacco legislation."

Much might be said in rebuttal of the claims made therein as to the comparatively innocuous character of tobacco. The argument, for example, that "the world-wide custom of using tobacco must serve some useful purpose as it can be taken for granted that useless or harmful habits do not survive in any species of animal," is hardly to be taken seriously, even from the point of view of logic, as proof of the harmlessness of tobacco. Must we "take it for granted" that the habit of hasty or imperfect mastication of food, or of living in ill-ventilated rooms, or of carelessly taxing the eyes in a poor light "serve some useful purpose" since they still "survive"?

But the writer desires particularly to refer to the studies of the effects of tobacco by Professor Meylan, of Columbia University, of which *American Medicine* says:

"Dr. Meylan has found that the students who use tobacco are taller, stronger and heavier than the abstainers, and that the difference is more than would be accounted for by the slightly greater age of the former. It proves that we were wrong in assuming that boys were stunted by the use of tobacco. * * * What a comment it all is upon our very human propensity to form theories before we know the facts. Hereafter, let all theorists present their facts first, and let the anti-cigarette laws wait until it is proved that they are needed."

It happens that there are some other important facts about these reported experiments, and it is but just to the public that they should be made known.

The Meylan experiments were described in the *New York Evening Post* early in March, the description being similar though more detailed than that of the *American Medicine* editorial. We are not informed as to the date in March on which this editorial on the experiments appeared in *American Medicine*, but it should be noted that Professor Meylan in a personal reply to a letter of inquiry from the writer, under a date in March as late as March 22d, said:

"I am carrying on a study as to the effects of tobacco on college men, but my work will not be completed for some weeks. When published, I shall send you a copy of the report. [Up to date, July 9th, it has not been received.] Most of the statements attributed to me in the newspapers as to the effects of tobacco originated in the minds of reporters who wanted to make a sensation."

So much for the authenticity and probable origin of the reports of the experiments which *American Medicine* says "prove that we were wrong in assuming that boys were stunted by the use of tobacco."

But did even the statements published, apparently unreliable as they were, prove this? The statistics credited to the Meylan investigations by the *Evening Post* were as follows:

*Columbia Smokers and Non-Smokers.*

| | Age— Yrs. | mos. | Height, cm. | Weight, kg. | Lung capacity, lit. | Strength, kg. |
|---|---|---|---|---|---|---|
| Smokers | 20 | 10 | 171.57 | 61.28 | 4.15 | 586 |
| Non-Smokers | 19 | 8 | 170.40 | 59.77 | 4.07 | 568 |
| Difference | 1 | 2 | 1.17 | 1.51 | .08 | 18 |

Any but the most superficial reader would ask at once: How much gain may the nineteen-year-old men ordinarily be expected to make during the year and two months that must elapse before they are as old as the smokers with whom they were compared?

*Amherst Students' Average Gain.*

An approximate answer may be found in the records of Professor Hitchcock of Amherst, who was the first to elaborate a system of measurements for college students. A table of measurements taken at Amherst in 1877-78 (before the use of tobacco had become as prevalent as now) showed that the average gain in height between the ages of nineteen and twenty was .732 inches; in weight, 2.67 pounds; in lung capacity, 5.56 cubic inches.

If we add this average gain made by the Amherst students in one year to the present measurements credited to the Columbia non-smokers, expressed in the equivalent English weights and measures, the result shows that by the time these non-smokers are as old as the smokers they may expect to be one-quarter of an inch taller, and to have two-thirds cubic inches more lung capacity, weight about the same.

*Yale Students' Average Gain.*

A more strictly comparable set of figures, because covering the same period of time, one year and two months, at practically the same age—one month older—is to be found in the following records of Yale students kept by Professor Seaver:

| | Age— yrs. | mos. | Height, cm. | Weight, kg. | Lung capacity, lit. |
|---|---|---|---|---|---|
| | 20 | 11 | 176.5 | 68.5 | 4.53 |
| | 19 | 9 | 173.0 | 63.5 | 4.22 |
| Difference....... | 1 | 2 | 3.5 | 5.0 | .31 |

If this difference be taken as the average rate of growth for 1 1-6 years to be expected at this age, and be added to the present status of the Columbia non-smokers (according to the newspaper statistics), it would give them when they reach the age of the present smokers, an advantage over the latter of .94 of an inch in height, 7.69 pounds in weight, and 14.36 cubic inches in chest capacity.

According to *American Medicine,* the reports admitted that some of the superiority claimed for the smokers was due to the fact that they were older, but, evidently, the editor did not figure out the measurements that would correspond to difference in age, or he would have seen that the smokers were really behind non-smokers in bodily development.

Another important item of which no mention was made is that according to the statistics published in the public press, it had taken the smokers on an average of one year and two months longer to reach the Freshman class than it did the non-smokers.

A large work on the deleterious effects of tobacco by E. L. Carvajal, M. D., has recently been published by the Mexican government "as a work of public utility." The author, who admits that he himself is a tobacco user, advises as a prophylactic measure strict enforcement of regulations prohibiting smoking in schools, public offices and other public places over which the police have control, as the habit of smoking is almost invariably acquired by imitation. He appeals to the authorities to repress the use of tobacco among boys, and to physicians to dispel the popular idea that the tobacco habit is harmless. In conclusion, he appeals to his "companions in slavery," saying, "Let us warn the innocent who sin from ignorance of the calamitous results of tobacco."

CORA FRANCES STODDARD,
Cor. Sec. The Scientific Temperance Federation.

Boston, Mass.

